# Does linear equating improve prediction in mapping? Crosswalking MacNew onto EQ-5D-5L value sets

**DOI:** 10.1007/s10198-020-01183-y

**Published:** 2020-04-16

**Authors:** Admassu N. Lamu

**Affiliations:** Department of Global Public Health and Primary Care, University of Bergen, Bergen, Norway

**Keywords:** MacNew, EQ-5D-5L, Economic evaluation, Mapping, QALY, Utility, Heart disease, I1, C1

## Abstract

**Purpose:**

Preference-based measures are essential for producing quality-adjusted life years (QALYs) that are widely used for economic evaluations. In the absence of such measures, mapping algorithms can be applied to estimate utilities from disease-specific measures. This paper aims to develop mapping algorithms between the MacNew Heart Disease Quality of Life Questionnaire (MacNew) instrument and the English and the US-based EQ-5D-5L value sets.

**Methods:**

Individuals with heart disease were recruited from six countries: Australia, Canada, Germany, Norway, UK and the US in 2011/12. Both parametric and non-parametric statistical techniques were applied to estimate mapping algorithms that predict utilities for MacNew scores from EQ-5D-5L value sets. The optimal algorithm for each country-specific value set was primarily selected based on root mean square error (RMSE), mean absolute error (MAE), concordance correlation coefficient (CCC), and r-squared. Leave-one-out cross-validation was conducted to test the generalizability of each model.

**Results:**

For both the English and the US value sets, the one-inflated beta regression model consistently performed best in terms of all criteria. Similar results were observed for the cross-validation results. The preferred model explained 59 and 60% for the English and the US value set, respectively. Linear equating provided predicted values that were equivalent to observed values.

**Conclusions:**

The preferred mapping function enables to predict utilities for MacNew data from the EQ-5D-5L value sets recently developed in England and the US with better accuracy. This allows studies, which have included the MacNew to be used in cost-utility analyses and thus, the comparison of services with interventions across the health system.

## Introduction

Coronary heart disease (CHD) is the leading cause of death and disability worldwide, particularly in Western countries. The total number of deaths from CHD increased by 19% over the most recent decade, from 7.96 million deaths in 2006 to 9.48 million deaths in 2016 [1]. The rising prevalence of CHD deaths will lead to increased demand for healthcare services. Resources for the prevention and treatment of CHD are limited and compete with demands from other disease areas and uses [2]. Consequently, there is a need for evaluating the cost-effectiveness of CHD interventions as compared to the competing use of resources in other disease groups.

In the cost-effectiveness appraisal of competing healthcare programmes across disease areas, there is a growing interest in estimating health outcomes on a generic metric, such as quality-adjusted life years (QALYs) [3]. To obtain the quality adjustment weight in the QALY, generic preference-based measures are used [4]. In many clinical trials, however, condition- or disease-specific non-preference-based measures commonly applied. This is mainly because these measures tend to identify disease-specific changes in health that might not be picked up by generic preference-based measures, though they may miss side effects and the impact on possible co-morbidities [3, 11]. Thus, in the absence of preference-based measures, the second-best alternative is to ‘crosswalk’, or ‘map’, disease-specific scores onto generic preference-based values to express health improvements in terms of QALY, which allows cross-study comparability.

Condition- or disease-specific measures assess the special states and concerns of diagnostic groups. The self-administered MacNew Heart Disease Quality of life Questionnaire (MacNew) is designed to evaluate how daily activities and physical, emotional, and social functioning are affected by CHD and its treatment [5]. CHD can last for longer periods and re-occur, impairing the ability to cope with daily life. While MacNew is suitable to measure CHD impact, it does not produce utility. In contrast, generic preference-based measures provide a utility weight for calculating QALY, which is useful for economic evaluations. Among preference-based measures, the EuroQoL five-dimensional questionnaire (EQ-5D) [8] is the most widely applied in cost-effectiveness analyses. The EQ-5D is also the preferred measure of the quality of life for health technology assessment in many European countries [6]. Such measures provide valuations on a 0 (being dead) to 1 (full health) scale. Health states valued less than 0 are also allowed. Two versions of EQ5D are available: the three-level (3L) and five-level (5L). The 5L is the modified version of 3L by adding two severity levels to address the ceiling and sensitivity concerns with the earlier 3L version [7]. Recently, 5L value sets are being developed in many countries [8, 9].

The MacNew has been mapped to the EQ-5D and other generic preference-based instruments [2]. However, the EQ-5D in the previous study was based on an interim value set, which was a ‘crosswalk’ between the earlier 3L version and the revised 5L descriptive system [10]. Thus, a revised mapping algorithm may be required with the publication of the directly elicited EQ-5D-5L value sets.

Studies revealed that regression-based mapping approaches usually under-predict high scores and over-predict low scores, because of regression to the mean [11]. Regression to the mean also expected to produce predicted values from mapping functions that have lower levels of variance than observed values [11, 12]. Thus, Fayers and Hays [12] have suggested the use of linking strategies such as simple linear equating, equipercentile equating, and item-response theory (IRT) methodologies as alternatives. While regression-based models attempt to predict the most likely true preference-based value set using the profile-based score, linking try to find the preference-based value set that is equivalent to the profile-based score by aligning the score distributions of the two scales [12]. Few mapping studies had applied regression-based approaches in combination with scale aligning; i.e., they first predicted utility, and then applied scale aligning between predicted and observed values [13, 14]. In the present study, a similar approach has been followed—first obtained predicted value sets via regression-based techniques and then used simple linear equating to force the predicted values to have the same mean and variance as the observed value sets.

In general, the objective of this study was to estimate the EQ-5D-5L value sets from the MacNew profile measure. More specifically, this paper has three important motivations. First, to update the existing mapping algorithms for MacNew that was recently published [2] using the directly elicited EQ-5D-5L value sets. Second, to examine whether mapping algorithms for the MacNew differ across countries, by employing two country-specific health state preferences; i.e., EQ-5D-5L value sets for the English and the US (United States). Lastly, this study makes important methodological contributions by investigating the relative merits of five regression models, and eventually linearly aligning the predicted values along the observed scales. Best practice for the reporting of mapping studies are followed, in line with ‘Mapping onto Preference-based measures reporting Standards (MAPS)’ [15].

## Methods

### Data

Data were obtained from a large international Multi-Instrument Comparison (MIC) study, which includes both EQ-5D-5L, and MacNew in addition to other instruments. The MIC study was an online survey administered in six countries in 2011/12: Australia, Canada, Germany, Norway, UK, and the US. Among the disease groups included in this comprehensive international study, the current paper is based on the CHD group (*n* = 943). There was no missing information on the data used in this study. However, considering the lack of direct control in the online survey, several edit procedures such as a comparison of duplicated questions, and removal of respondents whose recorded completion time shorter than 20 min were conducted to ensure the quality of data. For further details on data and respondent recruitment, see Chen et al. [2] and Richardson et al. [16].

### Measures of variables

The EQ-5D-5L consists of five dimensions each with five severity levels. The dimensions include mobility, self-care, usual activities, pain/discomfort and anxiety/depression, while the five severity levels constitute no problems, slight problems, moderate problems, severe problems and unable to/extreme problems. In this paper, the directly elicited EQ-5D-5L value sets from two countries (England, and the US) were applied [17, 18]. Both the English and the US value sets were published based on the EQ-VT approach. The scale length is quite different for the two countries: the worst health state or the ‘pits’ (55555) equals − 0.285 for the English value set and − 0.573 for the US.

The MacNew is designed to assess the patient’s feelings about how CHD affects daily functioning and contains 27 items, each with a seven-point Likert scale in decreasing severity [19]. Responses can be combined and a global health-related quality of life score was calculated as the average of the 27 item scores. The MacNew also covers three-domain scales: physical limitation domain scale (13-items), emotional function domain scale (14-items), and social function domain scale (13-items). Each domain includes overlapping items. The total score for each domain was calculated by summing responses across all items in that domain. Finally, each subscale summary scores were linearly transformed onto a 0–1 scale; 0 indicating the worst; and 1 the best possible health state [20].

### Statistical analyses and estimation

#### Exploratory data analysis

The precision of the mapping approaches relies on the extent of overlapping between the source and target instruments [11]. The Spearman’s rank correlations (*ρ*) between the MacNew domain scales and the EQ-5D-5L value sets were evaluated with a 95% confidence interval (CI) computed using 1000 bootstrap iterations.

Exploratory factor analysis (EFA) was also conducted to understand if the MacNew domain scales and EQ-5D-5L dimensions could be described by the same latent constructs or factors. The EFA was employed using iterated principal factors, which has been recommended as the preferred method of factor extraction [21]. An eigenvalue greater than 1 and the scree plot test were used as factor retention criteria [22, 23]. Although there is no consensus on a single standard threshold, factor loadings of 0.40 and above were considered “meaningful”, or at least salient [24], suggesting that MacNew domain scales and EQ-5D-5L dimensions were capturing the same underlying construct. Oblique-promax rotation of factors was applied to allow for a possible correlation between extracted factors.

#### Regression analysis

A direct mapping technique was applied by regressing the EQ-5D-5L value set onto the MacNew domain scores, such as physical, emotional and social. The squared term of each domain was explored. Furthermore, age and gender were considered as covariates to make mapping equations applicable to all datasets.

Here, five regression methods have been considered, as there was no single gold standard algorithm that would best predict the EQ-5D-5L value sets: ordinary least squares (OLS), generalized linear model (GLM), one-inflated beta (OIB) regression, fractional regression model (FRM), and robust MM-estimator (MM). In each regression model, the final predictors were retained only when they were statistically significant (i.e. *p* < 0.05). Predictors were also required to be logically consistent: poorer scores on a source instrument should lead to lower utility on the target instrument. Squared-terms were only considered if linear terms significantly contributed to the model.

OLS was considered, as it is the most commonly used method in mapping literature [11]. The GLM is a flexible generalization of OLS that allows our target variable (1) to have a non-normal error distribution, and; (2) to accommodate the non-linear relationship with the predictor variables (through the link functions) [25]. The logit link function with Gaussian family fit the data well, and hence applied in the estimation of GLM.

The FRM is a semi-parametric approach, which does not make any distributional assumption about an underlying structure used to obtain the outcome variable, but requires the correct specification of the conditional mean outcome [26, 27]. Given a vector of independent variables (*X*) and a dependent variable (*Y*), the FRM can be summarized as:

1 $$E\left( {y_{i} |x_{i} } \right) = \mu_{i} = G\left( {X\beta } \right)$$

where *G*(·) is a known nonlinear function satisfying 0 ≤ *G*(·) ≤ 1 and *β* is a vector of parameters to be estimated. The complementary log–log (cloglog) is the best alternative functional form for *G*(.) and used as a link function in EQ-5D-5L prediction.

The zero–one-inflated beta regression is a fully parametric regression, which is flexible and capable of modelling dependent variables restricted between 0 and 1 including zero and one [28]. As there is no zero response in the present study, a one-inflated beta (OIB) regression has been chosen to estimate Eq. (1). It estimates the probabilities of having 1 as a separate process from values between 0 and 1 [29]. Assuming *π*_1*i*_ is the probability that individual *i* is fully healthy (i.e., has observed health equal to 1), and *π*_01*i*_ = (1 − *π*_1*i*_) is the probability that the individual has impaired health (0 < *y*_*i*_ < 1) drawn from a beta distribution with mean *µ*_*i*_, then the overall mean of the predicted utility is given by:

2 $$E\left( {y_{i} } \right) \, = \left( {1 - \pi_{1i} } \right)\mu_{i} + \pi_{1i}$$

The mean response of the continuous beta distribution μ_i_ and the probability masses of 1 (*π*_1*i*_) were modelled directly with the same set of predictors using logit transformation and given by:

3a $$\text{logit}\left( {\mu_{i} } \right) = X\beta_{\mu } ;\quad {\text{i.e.}},\,\, \mu_{i} = \frac{{e^{{X\beta_{\mu } }} }}{{1 + e^{{X\beta_{\mu } }} }}$$

3b $$\text{logit}\left( {\pi_{1i} } \right) = X\beta_{1} ;\quad {\text{i.e.,}}\,\, \pi_{1i} = \frac{{e^{{X\beta_{1} }} }}{{1 + e^{{X\beta_{1} }} }}$$

where *β*_*µ*_ and *β*_1_ is a vector of unknown coefficients (including constants) to be estimated for the mean of continuous beta distribution *µ*_*i*_ (i.e., for 0 < *y*_*i*_ < 1) and the probability mass at 1 (i.e., for *y*_*i*_ = 1), respectively. The standard beta regression and the zero–one-inflated beta regression have been detailed elsewhere [28, 30].

In both FRM and OIB, the observed EQ-5D-5L utilities were initially normalized onto a 0–1 scale using linear-transformation [20, 31] before entering into the regression as the dependent variable. Finally, predicted EQ-5D-5L utilities were back-transformed to the original scale.

The MM-estimation is one of the robust regression estimation methods that is used when the distribution of residual is not normal or there are some outliers that affect the model [32]. The MM-estimation has been described elsewhere [33, 34].

#### Linear equating

Regression-based mapping models usually produce biased predictions due to regression to the mean [11, 12]. Simple linear equating can reduce this problem [12–14]. Linear equating involves a transformation of predicted scores from each of the proposed regression models linearly to have the same mean and standard deviation as the observed EQ-5D-5L value sets. Thus, given observed EQ-5D-5L value set and its predicted values (Pred), predicted linear equating (Pred_LE_) is given by:

4 $${\text{Pred}}_{{{\text{LE}}}} = \mu_{{{\text{Obs}} }} + \frac{{\sigma_{{{\text{Obs}}}} }}{{\sigma_{{{\text{Pred}}}} }}\left( {{\text{Pred}} - \mu_{{{\text{Pred}}}} } \right)$$

where *µ*_Obs_ and *σ*_Obs_ were the mean and standard deviation of the observed EQ-5D-5L value sets and *µ*_Pred_ and *σ*_Pred_ were the mean and standard deviation of the predicted EQ-5D-5L value sets obtained from the regression models. Following Hays et al. [13], predictions outside of the observed range were constrained to the nearest observed scale.

#### Predictive accuracy

The predictive performance of each model was assessed by the root mean square error (RMSE) and mean absolute error (MAE). Since raw values of RMSE and MAE are misleading to compare datasets and models with different units or scales, they are normalized by dividing both RMSE and MAE by the range of the observed data. Such normalized RMSE (NRMSE) and normalized MAE (NMAE) are non-dimensional that would allow reasonable comparison across models or measures with different scales. Furthermore, the performance of each model was assessed by the square of the correlation coefficient between the observed and predicted values (*r*^2^). The degree of absolute agreement between the predicted and the observed EQ-5D-5L was also assessed using Lin’s concordance correlation coefficient (CCC) [35]. Finally, scatter plots between the observed and predicted values were reported to visualize the predictive performance of each model.

#### Cross-validation

The best practice validation should be conducted on a different sample from the one used to generate the regression results. In the absence of external data, the second-best approach was performing cross-validation by splitting the existing data into estimation and validation samples via random selection procedures. In this study, the leave-one-out cross-validation (LOOCV) has been used to evaluate the model fit in out-of-sample data. Zhang and Yang [36] showed that LOOCV is typically the best modelling procedure in both bias and variance for the predictive performance estimation. In LOOCV, the estimation model is trained on all the data except for one data point and a prediction is made for that point. This procedure has been repeated for all data points. The average RMSE, MAE and predicted-*r*^2^ (Pred *r*^2^) from each iteration were calculated for comparison of the models’ predictive performance. Pred *r*^2^ is a better way to validate the predictive ability of the model, particularly in predicting future values [40]. All statistical analyses were conducted using Stata^®^ version 16.0 (StataCorp LP, College Station, Texas, USA).

## Results

The sample characteristics were presented in Table 1. The estimated EQ-5D-5L utilities varied in both the mean score and the range between the value sets of the two countries. In the CHD sample, the mean English EQ-5D-5L value set exceeded the US value set by nearly 0.05. Emotional subscale was the one with the lowest mean (SD) of 0.683 (0.192) among MacNew domains. The correlations between EQ-5D-5L value sets and MacNew domains were presented in Table 2. All MacNew domain scales produced relatively high correlation with the EQ-5D-5L value sets (*r* ≥ 0.63). The highest correlation was observed between ‘MacNew Global’ and the English value sets: 0.75 (95% CI 0.72–0.78).

**Table 1:** Sample characteristics (*n* = 943)

Variable	Mean (SD)/*n* (%)	Min	Max
EQ-5D-5L, mean (SD)			
English	0.804 (0.206)	− 0.185	1
US	0.753 (0.264)	− 0.447	1
MacNew domains, mean (SD)			
Emotional	0.683 (0.192)	0.036	1
Physical	0.716 (0.209)	0.077	1
Social	0.755 (0.207)	0.064	1
Global	0.711 (0.183)	0.103	1
Socio-demographics			
Age (in years), mean (SD)	59.760 (13.321)	18	93
Female, *n* (%)	338 (35.8)		
Country, *n* (%)			
Australia	149 (15.8)		
Canada	154 (16.3)		
Germany	152 (16.1)		
Norway	151 (16.0)		
UK	167 (17.7)		
US	170 (18.0)		

*SD* standard deviation, *EQ-5D-5L* EuroQol five-dimensional five-level questionnaire, *UK* United Kingdom, *US* United States

**Table 2:** Correlation coefficients between MacNew domain scales and EQ-5D-5L value sets

	English value set			US value set		
	*ρ*	95% CI		*ρ*	95% CI	
		Lower	Upper		Lower	Upper
Emotional scale	0.681	0.645	0.717	0.627	0.585	0.669
Physical scale	0.724	0.691	0.757	0.726	0.692	0.759
Social scale	0.701	0.666	0.736	0.687	0.650	0.725
Global scale	0.749	0.718	0.779	0.720	0.686	0.755

*ρ* Spearman correlation coefficient, *CI* bootstrapped confidence interval with 1000 iterations, *US* United States

**Fig. 1:**
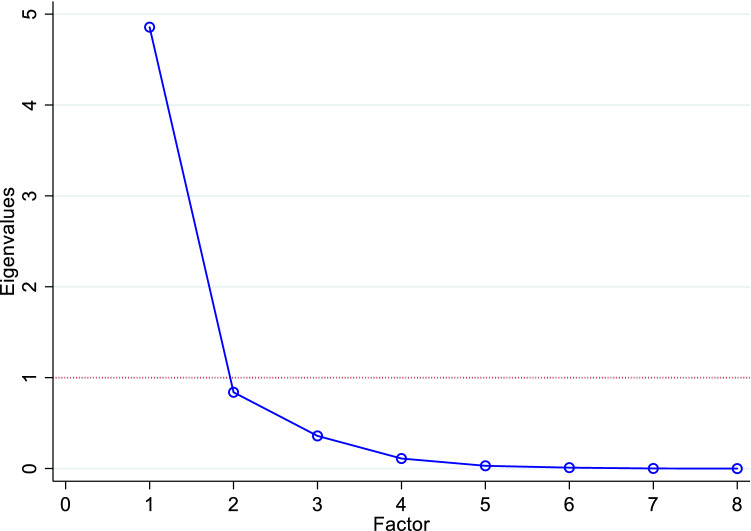
A scree plot showing the results of the iterated principal factor with one true factor underlying eight variables

The EFA was appropriate as indicated by a Kaiser–Meyer–Olkin (KMO) measure of sampling adequacy of 0.845 and a highly significant Bartlett’s Test of Sphericity ($\chi_{28}^{2}$ = 6633.465, *p* < 0.0001). The EFA produced one key factor with meaningful loadings on all MacNew domain scales, as well as all the five EQ-5D-5L dimensions. This overlap in the same factor suggests that the five EQ-5D-5L dimensions and the three MacNew domain scales would capture a similar latent construct. The result revealed adequate conceptual overlap between the source and target instruments such that the mapping algorithm would be valid. EFA results were detailed in Table 3 and Fig. 1.

**Table 3:** Exploratory factor analysis for the MacNew domain scales and EQ-5D-5L dimensions: iterated principal factor

Factor	Eigenvalue	Difference	Proportion	Cumulative
*Panel-A: unrotated factor loadings*				
Factor1	4.669	4.054	1.000	1.000
Factor2	0.615	0.468	0.132	1.132
Factor3	0.147	0.170	0.032	1.163
Factor4	− 0.023	0.101	− 0.005	1.158
Factor5	− 0.125	0.019	− 0.027	1.132
Factor6	− 0.144	0.030	− 0.031	1.101
Factor7	− 0.174	0.123	− 0.037	1.064
Factor8	− 0.297		− 0.064	1.000

Variable	Factor 1	Uniqueness
*Panel-B: rotated factor matrix*		
MacNew domain scales		
MacNew emotional	0.793	0.371
MacNew physical	0.904	0.183
MacNew social	0.910	0.172
EQ-5D-5L dimensions		
Mobility	0.736	0.459
Self-care	0.586	0.656
Usual activities	0.801	0.359
Pain/discomfort	0.705	0.503
Anxiety/depression	0.610	0.627

*EQ-5D-5L* EuroQol five-dimensional five-level questionnaire, *MacNew* MacNew Heart Disease Quality of life Questionnaire

**Fig. 2:**
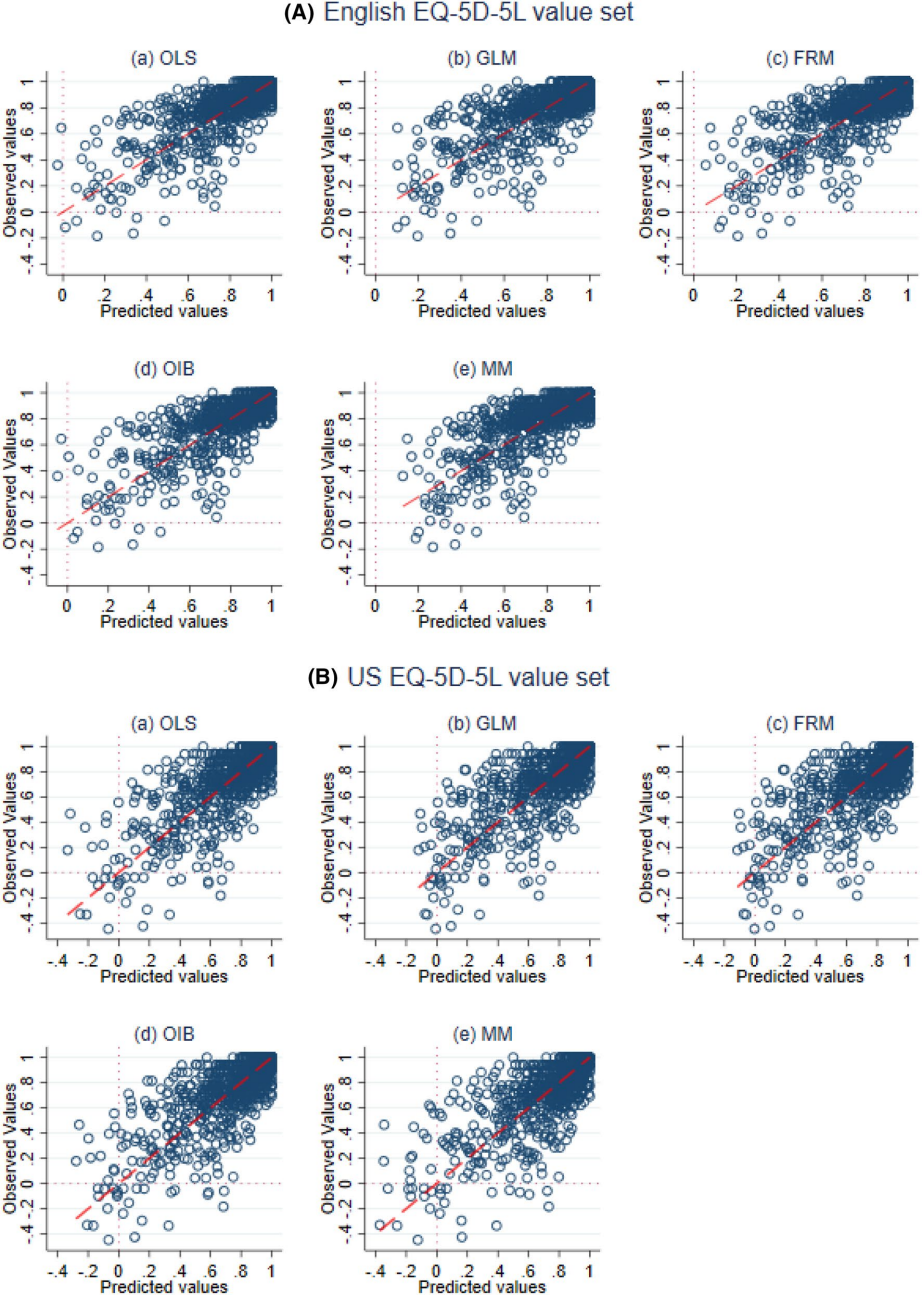
Scatter plots of observed vs predicted EQ-5D-5L value sets. *OLS* ordinary least square, *GLM* generalized linear model, *FRM* fractional regression model, *OIB* one-inflated beta regression. Broken line is a line along which observed and predicted value sets are equal

**Fig. 3:**
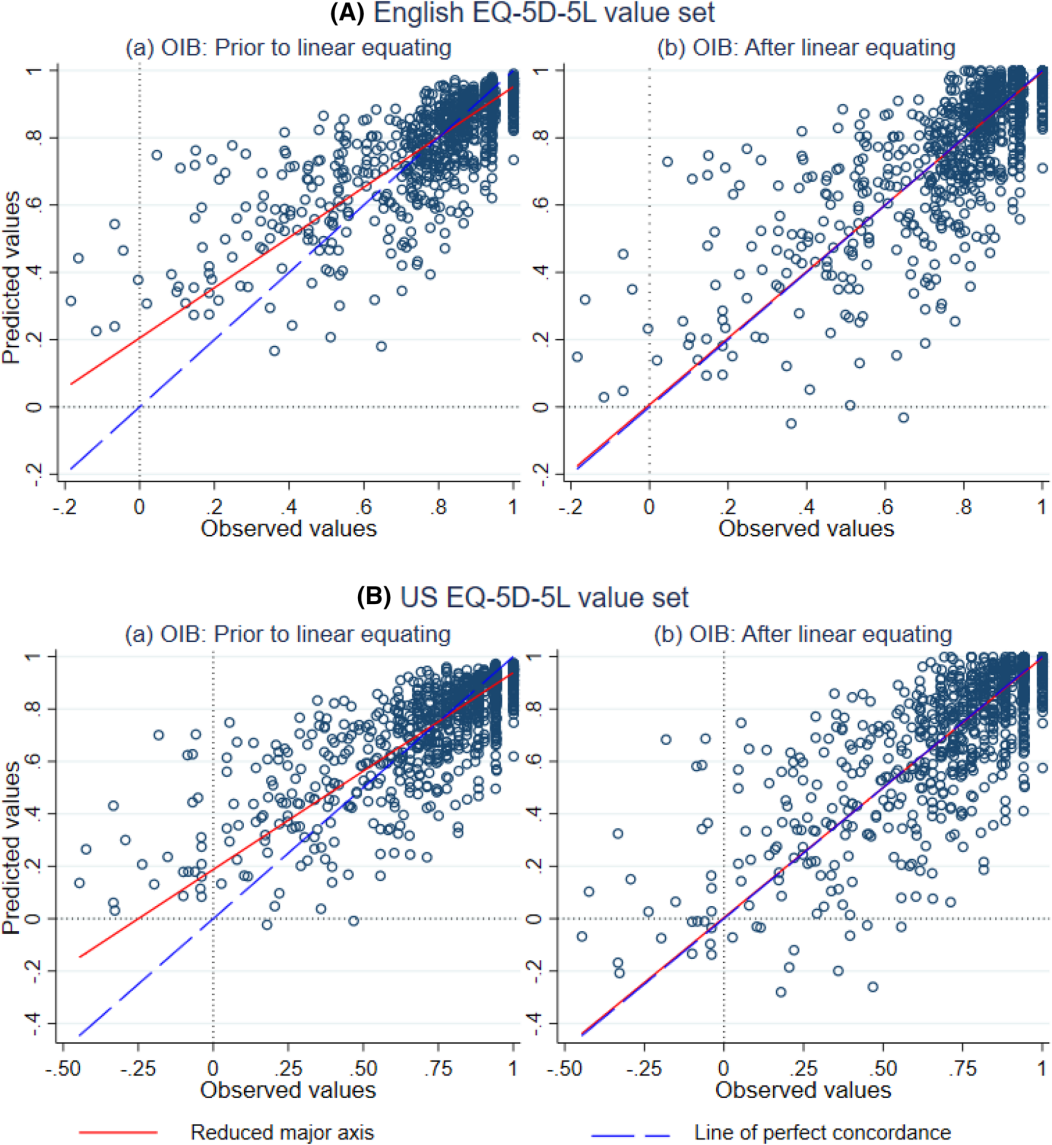
Scatter plot of predicted vs observed EQ-5D-5L value sets for the preferred model: upper panel for the English value set and lower panel for the US value set. *NB:* red line depicts reduced major axis (RMA) line, which shows a measure of the centre of the data; broken blue line is a line along which observed value sets equal predicted utilities. Perfect prediction occurs when RMA line and the line of perfect concordance overlaps. *US* United States, *OIB* one-inflated beta regression

Table 4 presented the performance of models assessed by four goodness-of-fit indicators. For both the English and the US value sets, OIB regression model consistently performed best in terms of all criteria. Interestingly, results from cross-validation supported the same model. The scatter plot also supported this result (Fig. 2). Both GLM and FRM performed well following OIB. When the English and the US value sets were compared in terms of raw RMSE and MAE, the English value set revealed superior predictive accuracy. However, after scale adjustment, both instruments have shown fairly similar predictive accuracy (see Fig. 3 and Table 4).

**Table 4:** Model performance in the prediction of EQ-5D-5L from the MacNew domain scales

	English						US					
Model	RMSE	MAE	NRMSE	NMAE	CCC	*r*^2a^	RMSE	MAE	NRMSE	NMAE	CCC	*r*^2a^
*Panel-A: Goodness-of-fit*												
OLS	0.1333	0.0922	0.1125	0.0778	0.7650	0.5845	0.1694	0.1188	0.1171	0.0821	0.7690	0.5920
GLM	0.1325	0.0909	0.1118	0.0767	**0.7680**	**0.5909**	0.1690	0.1181	0.1168	0.0816	0.7700	0.5932
FRM	0.1329	0.0919	0.1122	0.0775	0.7660	0.5866	0.1690	0.1183	0.1168	0.0818	0.7700	0.5934
OIB	**0.1323**	**0.0901**	**0.1116**	**0.0760**	**0.7680**	**0.5909**	**0.1684**	**0.1173**	**0.1164**	**0.0811**	**0.7720**	**0.5963**
MM	0.1607	0.0979	0.1356	0.0826	0.7560	0.5660	0.1770	0.1178	0.1223	0.0814	0.7580	0.5749
*Panel-B: Cross-validation*												
OLS	0.1341	0.0923	0.1131	0.0779	–	0.5773	0.1704	0.1183	0.1178	0.0818	–	0.5840
GLM	0.1333	0.0911	0.1125	0.0769	–	0.5819	0.1699	0.1183	0.1174	0.0818	–	0.5864
FRM	0.1334	0.0919	0.1126	0.0776	–	0.5812	0.1696	0.1184	0.1172	0.0818	–	0.5881
OIB	**0.1225**	**0.0836**	**0.1034**	**0.0705**	–	**0.5850**	**0.1689**	**0.1174**	**0.1167**	**0.0811**	–	**0.5917**
MM	0.1550	0.0958	0.1308	0.0808	–	0.5624	0.1771	0.1182	0.1224	0.0817	–	0.5716

Best results are in bold type

*RMSE* root mean squared error, *MAE* mean absolute error, *NRMSE* normalized root mean square error, *NMAE* normalized mean absolute error, *CCC* concordance correlation coefficient, *r*^*2*^ square of correlation coefficient between predicted and observed value sets, *OLS* ordinary least square, *GLM* generalized linear model, *FRM* fractional regression model, *OIB* one-inflated beta regression, *US* United States

^a^*r*^2^ in panel-B indicates predictive *r*^2^

The predictive accuracy of mapping algorithms at different distributions is illustrated in Table 5 (Panel-A). For the preferred model, the respective 5th, and 95th percentiles of the predicted English value set were 0.48, and 0.96 compared with 0.35, and 1 for the observed value set. Similarly, the 5th and 95th percentiles of the predicted US EQ-5D-5L value set were 0.32, and 0.95 against 0.18, and 1 for the observed value set, respectively. These results showed that the best-fitting model is over-predicting at severe health states and under-predicting at better health. Linear equating (reported in Panel-B of Table 5) fully eliminated under-prediction of high scores and substantially reduced over-prediction of low scores.

**Table 5:** Distributions of observed vs predicted EQ-5D-5L value sets at different severity levels

Model	Mean	SD	p1	p5	p10	p25	p50	p75	p90	p95	p99	IQR	Min	Max
*Panel-A: Observed vs predicted values*														
English														
Observed	0.804	0.206	0.084	0.354	0.516	0.752	0.866	0.942	1.000	1.000	1.000	0.190	–0.185	1.000
Predicted														
OLS	0.803	0.158	0.291	0.462	0.582	0.729	0.848	0.919	0.959	0.976	1.000	0.190	0.168	1.000
GLM	0.804	0.162	0.311	0.444	0.563	0.723	0.858	0.925	0.962	0.979	1.000	0.201	0.248	1.000
FRM	0.803	0.155	0.337	0.472	0.567	0.721	0.854	0.926	0.954	0.963	0.973	0.205	0.241	0.982
OIB	0.805	0.154	0.302	0.475	0.583	0.735	0.851	0.918	0.951	0.963	0.978	0.184	0.168	0.992
MM	0.855	0.087	0.621	0.686	0.729	0.798	0.871	0.925	0.956	0.968	0.984	0.128	0.569	1.000
US														
Observed	0.753	0.264	–0.153	0.180	0.370	0.666	0.844	0.940	1.000	1.000	1.000	0.274	–0.447	1.000
Predicted														
OLS	0.752	0.203	0.104	0.319	0.458	0.655	0.815	0.905	0.948	0.970	0.996	0.250	–0.084	1.000
GLM	0.753	0.200	0.166	0.307	0.425	0.662	0.829	0.906	0.935	0.945	0.957	0.244	0.093	0.966
FRM	0.753	0.201	0.151	0.323	0.440	0.646	0.822	0.911	0.946	0.957	0.971	0.265	0.041	0.980
OIB	0.753	0.199	0.116	0.321	0.458	0.665	0.815	0.902	0.942	0.953	0.968	0.237	–0.022	0.977
MM	0.789	0.189	0.130	0.367	0.533	0.714	0.856	0.924	0.952	0.963	0.978	0.210	–0.015	0.997
*Panel-B: Observed vs predicted values after linear equating*														
English														
Observed	0.804	0.206	0.084	0.354	0.516	0.752	0.866	0.942	1.000	1.000	1.000	0.190	–0.185	1.000
Predicted														
OLS	0.800	0.202	0.135	0.359	0.515	0.706	0.862	0.954	1.000	1.000	1.000	0.248	–0.026	1.000
GLM	0.801	0.202	0.181	0.349	0.500	0.702	0.872	0.957	1.000	1.000	1.000	0.254	0.102	1.000
FRM	0.802	0.205	0.184	0.364	0.490	0.694	0.872	0.966	1.000	1.000	1.000	0.272	0.056	1.000
OIB	0.802	0.204	0.131	0.363	0.507	0.710	0.866	0.956	0.999	1.000	1.000	0.246	–0.048	1.000
MM	0.795	0.196	0.251	0.405	0.507	0.669	0.842	0.970	1.000	1.000	1.000	0.302	0.128	1.000
US														
Observed	0.753	0.264	–0.153	0.180	0.370	0.666	0.844	0.940	1.000	1.000	1.000	0.274	–0.447	1.000
Predicted														
OLS	0.749	0.260	–0.090	0.189	0.370	0.626	0.834	0.951	1.000	1.000	1.000	0.324	–0.334	1.000
GLM	0.752	0.264	–0.023	0.163	0.320	0.632	0.852	0.954	0.992	1.000	1.000	0.322	–0.119	1.000
FRM	0.753	0.262	–0.018	0.167	0.323	0.634	0.853	0.954	0.992	1.000	1.000	0.321	–0.114	1.000
OIB	0.751	0.262	–0.095	0.177	0.360	0.636	0.836	0.951	1.000	1.000	1.000	0.315	–0.279	1.000
MM	0.752	0.264	–0.170	0.161	0.394	0.647	0.846	0.941	0.980	0.996	1.000	0.294	–0.373	1.000

*p1* 1st percentile, *p5 5*th percentile, …, *p99 99*th percentile, *SD* standard deviation, *IQR* inter-quantile range, *EQ-5D-5L* EuroQol five-dimensional five-level questionnaire, *MacNew* MacNew Heart Disease Quality of life Questionnaire, *OLS* ordinary least square, *GLM* generalized linear model, *FRM* fractional regression model, *OIB* one-inflated beta regression

The best-fitting regression results for both the English and the US country-specific value sets were presented in Table 6. Except for the social domain scale, other MacNew domain scales were significant (*p* < 0.05) predictors in all models. While gender and age were significant (*p* < 0.05) in predicting the continuous beta regression, only gender predicts the inflation part. The predicted EQ-5D-5L value sets from MacNew domain scales can be calculated using the results reported in Table 6. First, the mean (*μ*_*i*_) for the continuous beta regression (0 < *y*_*i*_ < 1) and the probability mass at 1 (*π*_1*i*_) were estimated by applying the logit transformation provided in expressions (3a) and (3b), respectively. Then, the estimated *μ*_*i*_ and *π*_1*i*_ were applied to Eq. (2) to estimate the overall mean of predicted EQ-5D-5L utilities. Finally, the predicted EQ-5D-5L utilities would be aligned on the same scale as the observed utilities using Eq. (4).

**Table 6:** Regression results predicting EQ-5D-5L from MacNew subscales for the preferred model: OIB

Variables	English	US
	*β* (SE)	*β* (SE)
*Beta regression*		
Emotional	1.876*** (0.199)	1.591*** (0.218)
Physical	2.176*** (0.172)	2.626*** (0.193)
Female	− 0.095** (0.043)	− 0.107** (0.048)
Age (in years)	− 0.008*** (0.002)	− 0.011*** (0.002)
Constant	− 0.552*** (0.161)	− 0.499*** (0.174)
*One-inflate*		
Emotional	4.437*** (1.070)	4.437*** (1.070)
Physical	7.592*** (1.471)	7.592*** (1.471)
Female	− 0.496** (0.242)	− 0.496** (0.242)
Constant	− 10.802*** (1.094)	− 10.802*** (1.093)

In each model, EQ-5D-5L was a target or dependent variable. Robust standard errors in parentheses

*OIB* one-inflated beta regression, *EQ-5D-5L* EuroQol five-dimensional five-level questionnaire, *MacNew* MacNew Heart Disease Quality of life Questionnaire, *β* estimated coefficients, *SE* standard errors for β

***p < 0.01, **p < 0.05, *p < 0.1

## Discussion

The use of the EQ-5D instrument in health economic evaluation has been increasing. However, the generic preference-based measures in key trials or studies may not be commonly used [3]. Thus, there is a need for mapping of disease-specific instruments onto the preference-based values sets. The present study developed mapping functions from the widely used CHD rating scale, the MacNew, onto two country-specific EQ-5D-5L value sets. This enables the potential application of these measures to population-based studies and economic evaluations.

The strength of the mapping function depends on the degree of conceptual overlap between the descriptive systems of the source and target instruments [3, 11]. The result revealed adequate conceptual overlap between the source and target instruments such that the mapping algorithm would be valid. However, the three MacNew domain scales are overlapping. For instance, emotional and physical domain scales include items relating to social interaction. The social domain contains all social items but also items relating to physical mobility and self-esteem. Consequently, the social functioning domain has shown either statistically insignificant estimates or logically inconsistent signs in the estimated coefficients for the prediction of both the English and the US EQ-5D-5L value sets.

In this mapping study, the merits of five regression models have been examined based on four goodness-of-fit criteria. OIB regression consistently performed best in predicting EQ-5D-5L utilities. Interestingly, the beta-binomial regression model performed best in predicting EQ-5D utilities in several other mapping studies [4, 37–39]. GLM generally produced the second-best on nearly all criteria, except MAE for the US value set where MM-estimator is the second-best. Essentially, GLM and OIB equally performed well on both CCC and* r*^2^ in predicting the English value set. FRM and GLM performed quite similar in the prediction of the US vale set. The novelty of the FRM and the OIB model is that they are more appropriate for data that is bounded and they accounted for the nonlinearity in the data.

A recent study by Chen et al. [2] has published mapping functions from MacNew onto six preference-based instruments including the EQ-5D using the same data set, which differs in several important aspects from the current study. The study by Chen and colleagues only considered three regression models (OLS, GLM and MM). The present study, however, considered two more analytical approaches, addressing the characteristics of the data such as problems of normality and non-linearity. Most importantly, while the present study employed the directly elicited EQ-5D-5L value sets, the study by Chen and colleagues used the interim value set, which was a “cross-walk” between the earlier three-level EQ-5D value set and the EQ-5D-5L descriptive system [10]. Therefore, the preferred models and their performance in terms of goodness-of-fit criteria were quite different. For instance, the preferred model for the English value set in this study produced RMSE, MAE, CCC, and r^2^ values of 0.1323, 0.0901, 0.7680 and 0.5909, respectively. In the study by Chen and colleagues, the preferred model for predicting EQ-5D was OLS; and MAE (0.1117), intraclass correlation (0.827) and *r*^2^ (0.552) were reported as goodness-of-fit criteria. In general, the discrepancy observed between the two studies may partly be attributable to differences in the target instrument used and partly due to the mapping functions employed, as well as variations in the additional covariates applied in predicting EQ-5D-5L utility values.

Mapping algorithms generally suffer from over-prediction for respondents in poor health and under-prediction for respondents in better health, mainly because of regression to the mean [11]. This phenomenon is detailed in Table 5, Panel-A. Linear equating can reduce the typical problem of under-prediction of high scores and over-prediction of low scores [12]. With linear equating, the smallest predicted values considerably dropped for both the English and the US value sets (see Table 5, Panel-B). Yet, there is an overestimation of scores for less than the 10^th^ percentile of the EQ-5D-5L value sets. This may be attributable to the strong decrements of preference weights of the EQ-5D-5L at severe health states only with few observations. Nevertheless, there is clearly an improved predictive accuracy after linear equating. In addition to mean values, linear equating forces the predicted values to have the same standard deviation as observed values, resulting in similar variability between the estimated values for the linear equating models and the observed values [14].

The present study has assessed the mapping functions for two different EQ-5D-5L value sets against MacNew scale. Clearly, different EQ-5D-5L value sets produce different utility scores, especially at the lower end. For instance, the observed scale in the current dataset is 1.185 (i.e., − 0.185 to 1) for the English value set, and 1.447 (i.e., − 0.447 to1) for the US value set. Therefore, the country-specific mapping function could be a better option to reflect the preference from a particular country. Considering the scale differences between the two countries’ value sets, the scale adjusted RMSE and MAE are also reported. The results are quite similar for the two countries, though the English value set has shown slightly better predictive ability in terms of both NRMSE and NMAE (Table 4). In contrast, the US value set slightly outperformed in terms of both CCC and *r*^2^. Such differences are expected, because of cultural as well as methodological variations. Although both value sets followed EQ-VT approach, the English value set is a hybrid-based that combines composite time-trade-off (cTTO) and discrete choice experiment (DCE), and the US value set is cTTO-based.

This study has a number of strengths. First, several mapping functions have been investigated, among which the OIB outperformed the rest. The OIB model has the ability to predict within the given range and allows a non-linear relationship between the dependent and predictor variables. Secondly, the predicted-*r*^2^ helps identify where the model provides a good fit for the existing data; more importantly, it also indicates how a regression model predicts responses for the new dataset [40]. Another key advantage of predicted *r*^2^ is its ability to prevent overfitting of a model. The wider the gap between conventional *r*^2^ and predicted-*r*^2^, the stronger is the problem of overfitting. In this study, the discrepancy between the predicted-*r*^2^ and the conventional *r*^2^ is trivial, indicating a good model fit. Thus, future mapping studies are encouraged to report predicted-*r*^2^ in cross-validation of the predictive accuracy of models. Thirdly, the application of linear equating minimizes mapping bias due to regression to the mean, which is a novel approach to align two measures on the same scale. Because the objective of this study was to map MacNew domain scales to the equivalent EQ-5D-5L value sets, predicted EQ-5D-5L value sets from each regression model were transformed linearly to have the same mean and standard deviation as the observed EQ-5D-5L value sets. Therefore, linking methods provide accurate prediction, particularly at the group level, which is the case in most economic evaluations that apply QALYs. Such linking produces the preference-based value sets that are equivalent to the condition- or disease-specific scores by aligning the score distributions of the two on similar scales [12]. In vein with other studies [13, 14, 29], the estimated EQ-5D-5L scores should be used only for group-level (not for the individual level) analysis.

With regard to study limitations, self-selection bias might have occurred, as respondents were volunteered to participate in the online survey. As generalizability is a major issue for mapping studies, the proposed mapping function should be tested on how the model performs in different CHD patient populations.

In conclusion, this study has developed a set of mapping algorithms to predict EQ-5D-5L value sets from the MacNew domain scales. Thus, in the absence of generic preference-based value sets, the preferred mapping model can adequately convert disease-specific scores onto a generic outcome metric like QALYs, which facilitates economic evaluations of CHD health interventions. The linear equating model may provide more accurate estimates of EQ-5D-5L utility values.
